# A mass spectrometry-based strategy for investigating volatile molecular interactions in microbial consortia: unveiling a *Fusarium*-specific induction of an antifungal compound

**DOI:** 10.3389/fmicb.2024.1417919

**Published:** 2025-02-25

**Authors:** Antonio Azzollini, Barbara Sgorbini, Nicole Lecoultre, Carlo Bicchi, Jean-Luc Wolfender, Patrizia Rubiolo, Katia Gindro

**Affiliations:** ^1^School of Pharmaceutical Sciences, University of Geneva, Geneva, Switzerland; ^2^Institute of Pharmaceutical Sciences of Western Switzerland (ISPSO), University of Geneva, Geneva, Switzerland; ^3^Department of Laboratory Medicine and Pathology, Lausanne University Hospital (CHUV), Lausanne, Switzerland; ^4^Department of Drug Science and Technology, University of Turin, Turin, Italy; ^5^Agroscope, Mycology Group, Nyon, Switzerland

**Keywords:** volatile molecules, mass spectrometry, co-culture, VOC-mediated interactions, *Fusarium*, antifungal, *Aspergillus*, *Cladosporium*

## Abstract

Co-cultivation of microorganisms has emerged as a promising methodology for deciphering the intricate molecular interactions between species. This approach facilitates the replication of natural niches of ecological or clinical relevance where microbes consistently interact. In this context, increasing attention has been addressed toward elucidating the molecular crosstalk within fungal co-cultures. However, a major challenge in this area of research is determining the fungal origin of metabolites induced in co-cultivation systems. Molecules elicited in co-cultures may not be detectable in the individual cultures, making it challenging to establish which microorganism is responsible for their induction. For agar-diffused metabolites, imaging mass spectrometry can help overcome this obstacle by localizing the induced molecules during fungal confrontations. For volatile metabolites, however, this remains an open problem. To address this issue, in this study, a three-head-to-head co-culture strategy was developed, specifically focusing on the exploration of volatile interactions between fungi via headspace solid-phase microextraction combined with gas chromatography mass spectrometry. This methodology was applied to study the volatile molecular interactions of three fungal species: *Fusarium culmorum*, *Aspergillus amstelodami*, and *Cladosporium cladosporioides*. The adopted strategy revealed a *Fusarium-*specific induction of three volatile molecules: *γ-*terpinene and two unidentified sesquiterpene compounds. Interestingly, *γ-*terpinene showed antifungal activity in a bioassay against the other two fungal species: *Aspergillus amstelodami* and *Cladosporium cladosporioides*. The proposed methodology could help to investigate volatile molecular interactions and highlight metabolite induction specific to a particular fungus involved in *in vitro* fungal confrontations. This is relevant for a better understanding of the complex biosynthetic responses of fungi in consortia and for identifying volatile molecules with antifungal activity.

## 1 Introduction

Microorganisms play an essential role in sustaining life in both plant and animal organisms (Zilber-Rosenberg and Rosenberg, 2008; Singh et al., 2020; Suman et al., 2022). Microbes interact with each other and their environment to form complex ecosystems, forming intricate networks of ecological relationships that are essential to ecosystem functioning (Moënne-Loccoz et al., 2015; Cosetta and Wolfe, 2019; Gralka, 2023). Their interactions can be mediated by sophisticated molecular communication that regulates key processes such as competition, cooperation, and symbiosis (Braga et al., 2016; Netzker et al., 2020; Weiland-Bräuer, 2021). A thorough understanding of these molecular dynamics is crucial for deciphering the functioning of microbial consortia and their impact not only on the health of host organisms but also on the ecological balances in which they are involved (Rapp et al., 2020; Kapoore et al., 2022; Liu and Xu, 2022).

In this context, microbial co-cultivation has emerged as a promising strategy for the activation of silent biosynthetic clusters (genes that are typically dormant under standard laboratory or environmental conditions) and the stimulation of the production of new bioactive molecules. Furthermore, this approach has the potential to facilitate the study of the intricate molecular dialog among different microbial species, including fungi (Tan et al., 2019; Liu and Kakeya, 2020; Xu et al., 2023). In practice, this strategy involves *in vitro* co-cultivation of microorganisms in a confined environment (e.g., a Petri dish) (Arora et al., 2020; Selegato and Castro-Gamboa, 2023). In particular, this technique has been employed to study fungal-fungal interactions, including studies between different endophytic fungi (Chagas et al., 2013; Li et al., 2020), as well as interactions involving fungi from the marine environments (Wang et al., 2015; Ding et al., 2017) and from the extreme environments (Stierle et al., 2017). The co-cultivation of fungi has enabled the synthesis of numerous structural classes, including alkaloids (Zhu and Lin, 2006; Zhu et al., 2011; Zhu et al., 2013; Afiyatullov et al., 2018; Sadahiro et al., 2020), terpenes (Zhou et al., 2018), polyketides (Chagas et al., 2013; Li et al., 2020) and peptides (Huang et al., 2014; Li et al., 2014). Moreover, these metabolites showed a broad spectrum of biological properties, including antifungal (Chagas et al., 2013), antibacterial (Zhu et al., 2011), and antiviral activities (Yang et al., 2018; Knowles et al., 2022).

Fungal co-cultures can be cultivated on solid media in Petri dishes ranging from 9 to 15 cm in diameter (Bertrand et al., 2014b). This setup requires protracted extraction and sample preparation phases, leading to significantly prolonged experimentation durations. In this regard, for a more efficient experimental setting, the miniaturization of fungal co-cultivation has proven to be an appropriate way to rapidly collect metabolite profiles from an extensive set of replicates required for metabolomic studies. For example, multi-well 2-cm Petri dishes have been used to co-cultivate *Fusarium* and *Aspergillus* strains and highlight their dynamic agar-diffused molecular crosstalk (Bertrand et al., 2014a). To investigate fungal interactions also at the volatile level, this experimental methodology was adapted to a miniaturized setup based on the co-cultivation of fungi directly in 20-mL headspace vials, which were then sampled via headspace solid-phase microextraction (HS-SPME). This approach was used to study an ecologically relevant fungal co-culture of *Eutypa lata* (Pers.) Tul. & C. Tul. (Diatrypaceae) and *Botryosphaeria obtusa* (Schwein.) Shoemaker (Botryosphaeriaceae) and revealed dynamically induced molecules at both volatile and non-volatile levels (Azzollini et al., 2018).

Overall, in the field of fungal co-cultures, numerous investigations have been conducted to identify induced metabolites using liquid chromatography mass spectrometry (LC–MS)-based methodologies (Bertrand et al., 2013; Bohni et al., 2016; Xu et al., 2018; Murakami et al., 2020). Such strategies enable MS-guided purification of selected metabolites, possibly streamlining the complete identification of targeted molecules (Azzollini et al., 2016).

Unlike LC–MS-based approaches, which have been broadly used for the profiling of non-volatile compounds, limited studies have explored the use of gas chromatography–mass spectrometry (GC–MS) for the profiling of fungal volatile molecules (Knowles et al., 2022).

Fungi are abundant producers of various volatile organic compounds (VOCs) encompassing a diverse range of chemicals, such as aliphatic and aromatic hydrocarbons, aldehydes, ketones, esters, alcohols, and mono-, sesqui-, and diterpenes (Effmert et al., 2012; Kramer and Abraham, 2012). Interestingly, certain VOCs emitted by fungi display antifungal and antibacterial properties, thereby underlining their potential biological relevance (Hung et al., 2015; Inamdar et al., 2020; Karsli and Şahin, 2021). Fungal VOCs may play a role in influencing interactions within microbial consortia, influencing not only fungal-fungal interactions but also cross-kingdom relationships, such as those between fungi and bacteria (Khalid and Keller, 2021; Zhou et al., 2022), plants (Russo et al., 2022; Razo-Belmán et al., 2023), and even human hosts (Inamdar et al., 2020; El Jaddaoui et al., 2023). For example, in plant-fungal interactions, VOCs from fungi such as *Trichoderma* can promote plant growth, illustrating their possible role in plant-fungal ecosystems (Lee et al., 2016; You et al., 2022). From a human health standpoint, the study of fungal VOCs is of particular importance in the light of the growing recognition of the lung and oral human mycobiome (Ghannoum et al., 2010; Nilsson et al., 2019; Tiew et al., 2020). In regions where gas-phase interactions are possible, such as the oral cavity and lungs, fungal VOCs may play a role in shaping both local microbial ecology and influencing human health (Nguyen et al., 2015; Kong and Morris, 2017; Vitte et al., 2022). Overall, the variation of the emission of VOCs during fungal interactions has been documented (Guo et al., 2019; Speckbacher et al., 2021; Escudero-Leyva et al., 2023), however, investigation of VOC production within fungal co-cultures remains an emerging field, opening a vast landscape for further scientific investigations.

Furthermore, a critical challenge associated with investigation of volatile interactions in fungal co-cultures is that volatile metabolites detected in the co-culture, but not in the corresponding single cultures (*de novo* induced), cannot be linked to any of the two interacting fungi (Bertrand et al., 2014b; Arora et al., 2020). Thus, the species producers of these metabolites remain unknown. For agar-diffused molecules, imaging mass spectrometry can provide a valuable solution by accurately localizing the induced molecules involved in interspecies interactions (Watrous and Dorrestein, 2011; Sica et al., 2014; Müller et al., 2022). However, this remains an unresolved issue for volatile molecules induced during microbial confrontations. Accurate identification of the fungus responsible for the induction of volatile molecules is essential to deepen our understanding of the ecological dynamics and behavior of fungi within a particular co-culture or community. Therefore, in this study, a strategy was developed to address this issue.

In particular, this study investigated three fungal species: *Cladosporium cladosporioides* (Fresen.) G.A. de Vries, *Aspergillus amstelodami* (L. Mangin) Thom & Church and *Fusarium culmorum* (Wm. G. Sm.) Sacc. These fungi have been selected as they have been possibly associated with the human oral mycobiome (Ghannoum et al., 2010), where gas-phase interactions relevant to both human microbiota and host physiology can take place (Hertel et al., 2018; Monedeiro-Milanowski et al., 2022; Kiss et al., 2023; Weber et al., 2023).

As part of this work and as a proof-of-concept, these three fungal species were studied in a co-cultivation system that enabled sampling of the metabolome headspace and allowed for the identification of species-specific inductions in a three-head-to-head confrontation model.

A specific bioassay was used to evaluate the biological activity of an identified induced molecule and to understand its potential role within the fungal consortia (Azzollini et al., 2018). This bioactivity test was designed to allow only the migration of volatile compounds within the test environment.

## 2 Materials and methods

### 2.1 Chemicals

Potato Dextrose Agar (PDA, Difco) was used as the culture medium. The pure reference standard of *γ*-terpinene and the solvent dibutylphthalate (DBP) were purchased from Sigma–Aldrich.

### 2.2 Biological material

Three fungal species were selected for this study. The strains were purchased from the collection of CBS (Central Utrecht, The Netherlands): specimen numbers 119376, 134912, 120098, respectively, *Aspergillus amstelodami*, *Cladosporium cladosporioides* and *Fusarium culmorum*. These three fungal strains were stored at 4°C in the Agroscope dynamic mycotheca in vials containing a diluted potato dextrose broth solution (1:4) (Sigma-Aldrich).^1^

### 2.3 Experimental settings of culture and co-culture conditions

*Fusarium culmorum*, *Aspergillus amstelodami,* and *Cladosporium cladosporioides* pure cultures were prepared by placing 2-mm PDA plugs of fungal pre-cultures in the center of the headspace vial. The co-cultures were prepared by placing two 2-mm agar plugs of a pre-culture of the two different fungal species on opposite sides of the headspace vial (Supplementary Figure S1). Immediately after inoculation, each vial was closed with an appropriate cap (HDSP cap 18 mm magnetic PTFE/Sil, Agilent Technologies, United States). Blank samples (PDA only) were prepared. Cultures were incubated at 21°C in the dark. To study the induction of volatile molecules in these three fungal co-cultures, nine replicates (*n* = 9) of each single culture and co-culture were sampled via HS-SPME and analyzed via GC–MS after 9 days of incubation.

### 2.4 Analysis of the volatile fractions using HS-SPME-GC-MS

The volatile fraction (headspace) of each individual single culture and co-culture was sampled using a 2-cm DVB/CAR/PDMS SPME fiber (Supelco, Bellefonte, PA, United States) introduced directly into the headspace vial used for cultivation. Sampling was performed using an MPS2 autosampler (Gerstel, Mülheim a/d Ruhr, Germany) at 40°C (no pre-equilibrium, 30 min of sampling). The fiber was automatically injected into an Agilent 7890 GC coupled to an Agilent 5975C MS (Agilent, Little Falls, DE, United States). Internal standard [C13 diluted in dibutylphthalate (DBP)] was preloaded onto the fiber using the in-fiber standardization technique (Wang et al., 2005). Sampling was performed only once for each culture to avoid any perturbation of the volatile fraction over time. The GC–MS conditions were as follows: inlet temperature 250°C, desorption time: 5 min, split injection (1/10 split ratio), carrier gas (helium at a constant flow rate of 1 mL/min); column: HP5MS (5% phenyl, 95% polysiloxane - 30 m × 0.25 mm i.d. × 0.25 μm; Agilent Technologies). Oven temperature program: 50°C (1 min) - 3°C/min - 200°C - 15°C/min - 250°C (4 min). MS was operated in the EI mode at 70 eV with a mass range of 35–350 amu in full scan mode.

### 2.5 GC-MS data processing and deconvolution of GC peaks

Data were processed using an Agilent MSD ChemStation version F.01.03.2357. GC peaks were identified by comparing their linear retention indices (calculated *versus* a C9-C25 hydrocarbon mixture) and their mass spectra in comparison with those present in commercially available libraries (Wiley, Adams, NIST). Standard deviation was employed in the evaluation of normalized area calculations for induced volatile molecules. *γ*-Terpinene identity was confirmed by analyzing the pure reference standard (Sigma-Aldrich).

### 2.6 Bioassay

To evaluate the antifungal activity of the volatile induced compound γ-terpinene, a 9-cm Petri dish, divided in two by a plastic septum, was used. This partitioning allowed only the diffusion of volatile molecules between the two sectors of the dish. Distinct pure cultures of *Aspergillus amstelodami* and *Cladosporium cladosporioides* were inoculated on PDA within the first sector of the Petri dish using a 2-mm agar plug from their respective pre-cultures. The compound γ-terpinene was injected into a filter paper posed in the second sector of the Petri dish. The activity of γ-terpinene was assessed at the following concentrations: 917 μL/L, 524 μL/L, and 131 μL/L. The concentrations reported for this bioassay were expressed as liquid volume of each volatile compound per dish volume (for simplification, the dish volume was calculated without considering the plastic septum). For the control samples, no compound was injected into the second sector of the Petri dish. Mycelial growth was monitored after 7 days. Five replicate experiments were performed for each concentration.

## 3 Results and discussion

### 3.1 Implementation of a three-head-to-head co-culture strategy to highlight species-specific induction of VOCs

**Figure 1:**
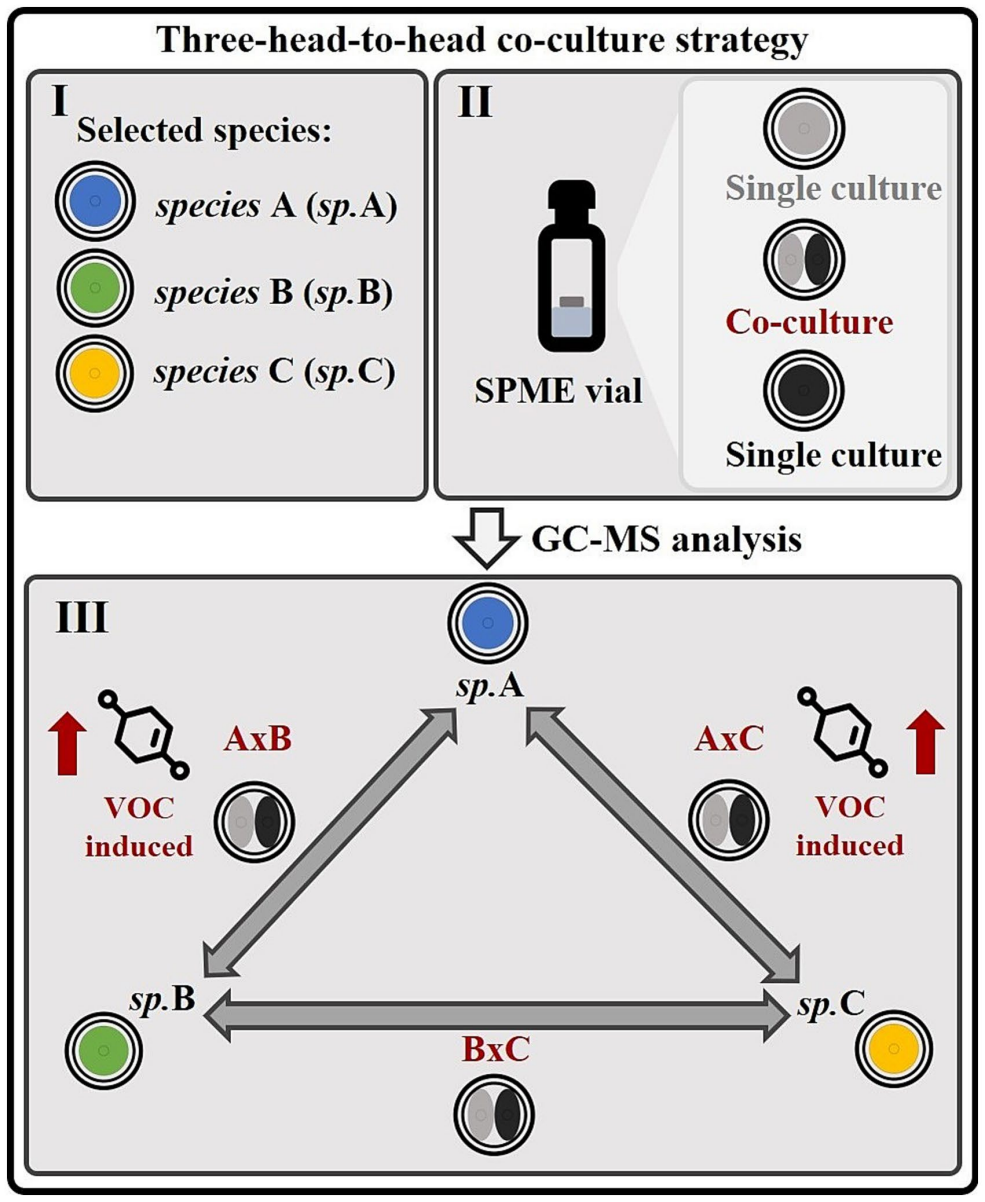
Strategy for investigating volatile interactions and species-specific metabolite induction in microbial or fungal co-cultures. (I) This experimental strategy is designed to investigate volatile interactions among three microbial or fungal species (A, B, C) and identify the species responsible for inducing specific metabolites within co-cultures. (II) The organisms are cultivated directly in SPME vials, allowing sampling of the volatile metabolome. (III) Three distinct co-cultures (A × B, A × C, and B × C), constituting a “head-to-head” system, as well as their corresponding monocultures, are analyzed using GC–MS. This setup enables the identification of species-specific inductions of volatile molecules. For instance, if a particular metabolite is detected in co-cultures A × B and A × C, but not in B × C, as illustrated in this figure, it can be inferred that species A is responsible for inducing the production of that volatile organic compound (VOC).

In the context of this study, a strategy has been devised for the purpose of investigating the volatile interactions that are mediated by VOCs (volatile organic compounds) in fungal co-cultures, and for identifying the fungus responsible for inducing volatile molecules within such co-cultures. This approach involves culturing both individual cultures and co-cultures in 20 mL SMPE vials within a three-head-to-head co-cultivation system (Figure 1). The fungal co-cultures and single cultures were further analyzed via gas chromatography–mass spectrometry (GC–MS) to investigate the volatile fractions of these samples. This experimental design was conceived to highlight species-specific induction of secondary metabolites, as in all confrontations, one partner is always opposed to the others in separate head-to-head co-cultures. For example, if a particular metabolite is induced in the co-culture of species A with B (AxB) and in the co-culture of species A with C (AxC), but absent in the co-culture of species B with C (BxC), this suggests that species A is responsible for inducing the molecule in question (Figure 1). This setup allows for the precise identification of the fungal species responsible for the induction of volatile secondary metabolites in a co-culture environment, facilitating the understanding of species-specific molecular interactions.

**Figure 2:**
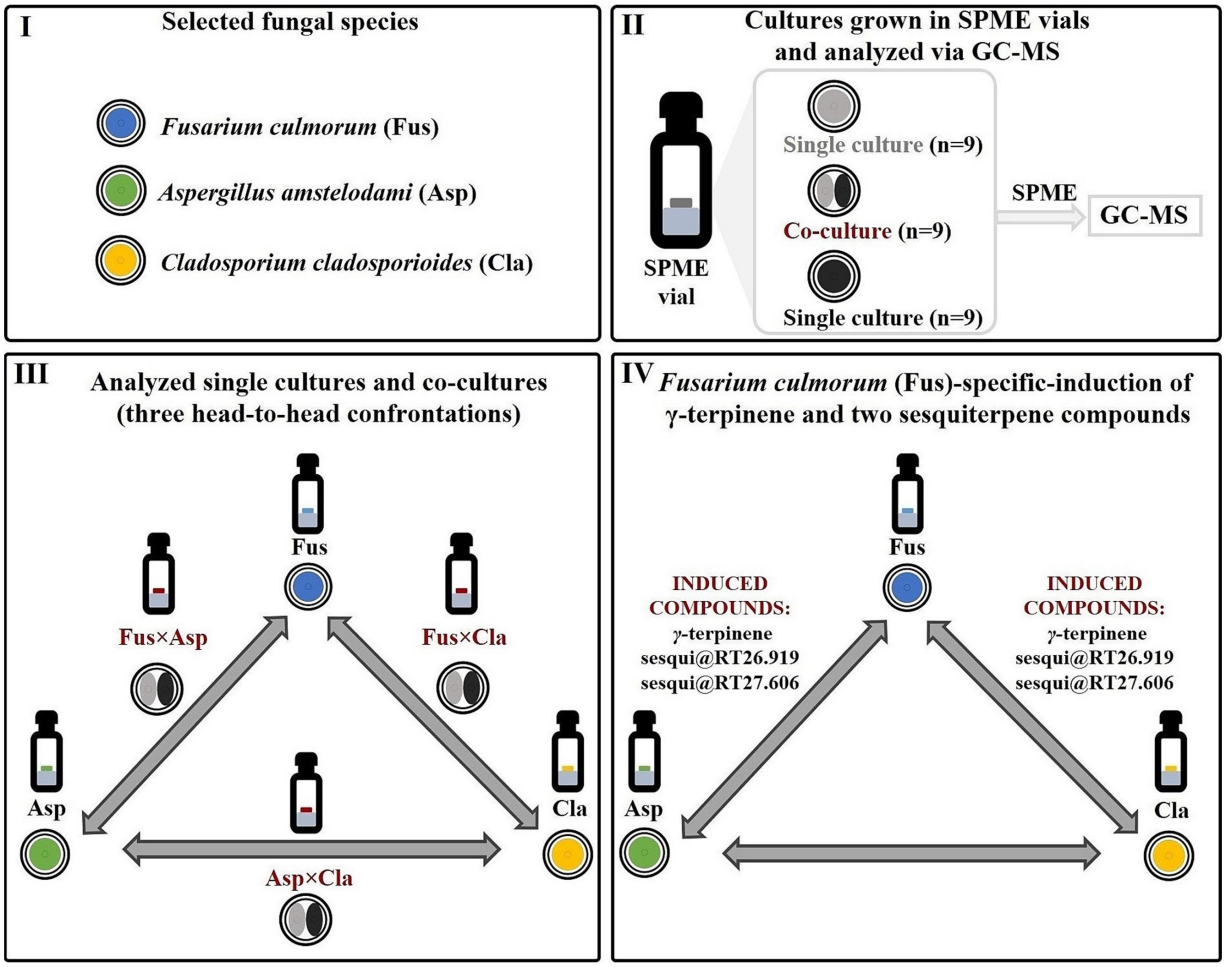
Implementation of the proposed strategy and detection of *Fusarium*-specific induction of *γ-*terpinene and two unidentified sesquiterpene compounds. (I) Volatile molecular interactions between three fungal species (*Fusarium culmorum, Aspergillus amstelodami* and *Cladosporium cladosporioides*) were investigated. (II) Fungal co-cultures, as well as the respective single cultures, were grown directly in SPME (headspace) vials, sampled using HS-SPME and analyzed using GC–MS. For each head-to-head fungal confrontation experiment, nine co-cultures, as well nine single cultures of each of the confronting fungi were studied. (III) Three different fungal co-cultures (constituting a three-head-to-head system) were analyzed: *Fusarium culmorum* vs. *Aspergillus amstelodami, Fusarium culmorum* vs. *Cladosporium cladosporioides*, *Aspergillus amstelodami* vs. *Cladosporium cladosporioides* (respectively referred to as FusxAsp, FusxCla and AspxCla) and of the corresponding single cultures (Fus, Asp, and Cla). (IV) A *Fusarium-*specific induction of the three volatile induced molecules (*γ*-terpinene, sesqui@RT26.919, and sesqui@RT27.606) was highlighted. It was observed that these molecules were elicited only in co-cultures where *Fusarium culmorum* (Fus) is one of the confronting fungi (FusxAsp and FusxCla). No metabolite induction was observed in the *Aspergillus amstelodami* vs. *Cladosporium cladosporioides* (AspxCla) co-cultures.

In this work, the aforementioned methodological approach was employed to investigate the volatile interspecies molecular crosstalk among *Fusarium culmorum* (Fus), *Aspergillus amstelodami* (Asp), and *Cladosporium cladosporioides* (Cla). These microbial fungi were selected because, interestingly, they may be related to the human oral mycobiome, where gas-phase interactions that are relevant to host physiology may occur (Ghannoum et al., 2010; Monedeiro-Milanowski et al., 2022; Kiss et al., 2023). In addition, *Fusarium* spp., *Aspergillus* spp., and *Cladosporium* spp. have also been notably associated to lung and respiratory health (Carneiro et al., 2011; Richardson et al., 2019; Ma et al., 2021). The aforementioned strains were grown in 2-mL of solid media in SPME (headspace) vials. To improve the statistical robustness of the experimental design, nine replicates of the three different co-cultures (*Fusarium culmorum* vs. *Aspergillus amstelodami, Fusarium culmorum* vs. *Cladosporium cladosporioides*, *Aspergillus amstelodami* vs. *Cladosporium cladosporioides*, respectively referred to as FusxAsp, FusxCla and AspxCla) and of the corresponding single cultures (Fus, Asp, Cla) were incubated for 9 days and sampled at this time point via HS-SPME (Figure 2). All experiments were performed concomitantly and under identical culture conditions. Cultivation in a 20-mL headspace vial was found to be appropriate for the analysis of the volatile metabolome in multiple confrontation studies (Azzollini et al., 2018). The two fungal species were simultaneously co-cultured in headspace vials to analyze variations in the volatile metabolome resulting from their interactions. This format allows for the rapid generation of a large number of replicates. Moreover, this cultivation setup is specifically designed to identify species-specific secondary metabolite induction, as each confrontation pairs one fungal species with another in the aforementioned three-head-to-head co-cultivation system. For instance, metabolite induction in FusxAsp and FusxCla co-cultures, with absence in AspxCla, can suggest a *Fusarium-*specific expression of the elicited molecule (Figure 2).

After 9 days of growth, each single culture and co-culture was sampled and analyzed using HS-SPME-GC–MS. Volatile compounds released into the headspace by the single cultures and co-cultures were sampled by introducing a SPME fiber, which allowed their accumulation on the fiber polymer at the end of the incubation period. Direct thermal desorption of the SPME fiber into the GC injector provided GC–MS profiling of the metabolites from both the single culture and co-culture volatile fractions.

### 3.2 Detection of a *Fusarium*-specific induction of *γ*-terpinene and two sesquiterpene compounds

After the GC–MS analysis of all single cultures and co-cultures, the compounds detected in the HS-SPME-GC–MS profiles were identified by comparing the acquired spectra with those of commercial libraries (Wiley, Adams, Nist) or by injecting a reference standard and using linear retention indices calculated *versus* a mixture of C9-C25 alkanes. The GC peaks of the total ion chromatogram were integrated, and the obtained areas were used to compare the profiles, thereby highlighting features induced in co-cultures that were not detected in single cultures. Three features were detected as induced in all the nine replicates of the two different co-cultures of FusxAsp and FusxCla: *γ-*terpinene and two unidentified sesquiterpene compounds.

The identity of the *γ-*terpinene was confirmed by co-injection of a commercially available standard (Supplementary Figure S2). The two unidentified sesquiterpene compounds @RT 26.919 and @RT 27.606 had both a molecular weight of 204 Da and a retention index of 1,413 and 1,431, respectively. In the absence of available standards and reference spectra in the available EI-MS libraries, it was not possible to achieve full *de novo* identification of these compounds. Furthermore, the scale at which the HS-SPME-GC–MS experiments were conducted did not permit the isolation and subsequent structural elucidation of these molecules via NMR. These two sesquiterpene compounds were referred to as sesqui@RT26.919 and sesqui@RT27.606, respectively, and only the compound type could be attributed based on the fragmentation pattern of their associated EI-MS spectra (see Supplementary Figure S3).

**Figure 3:**
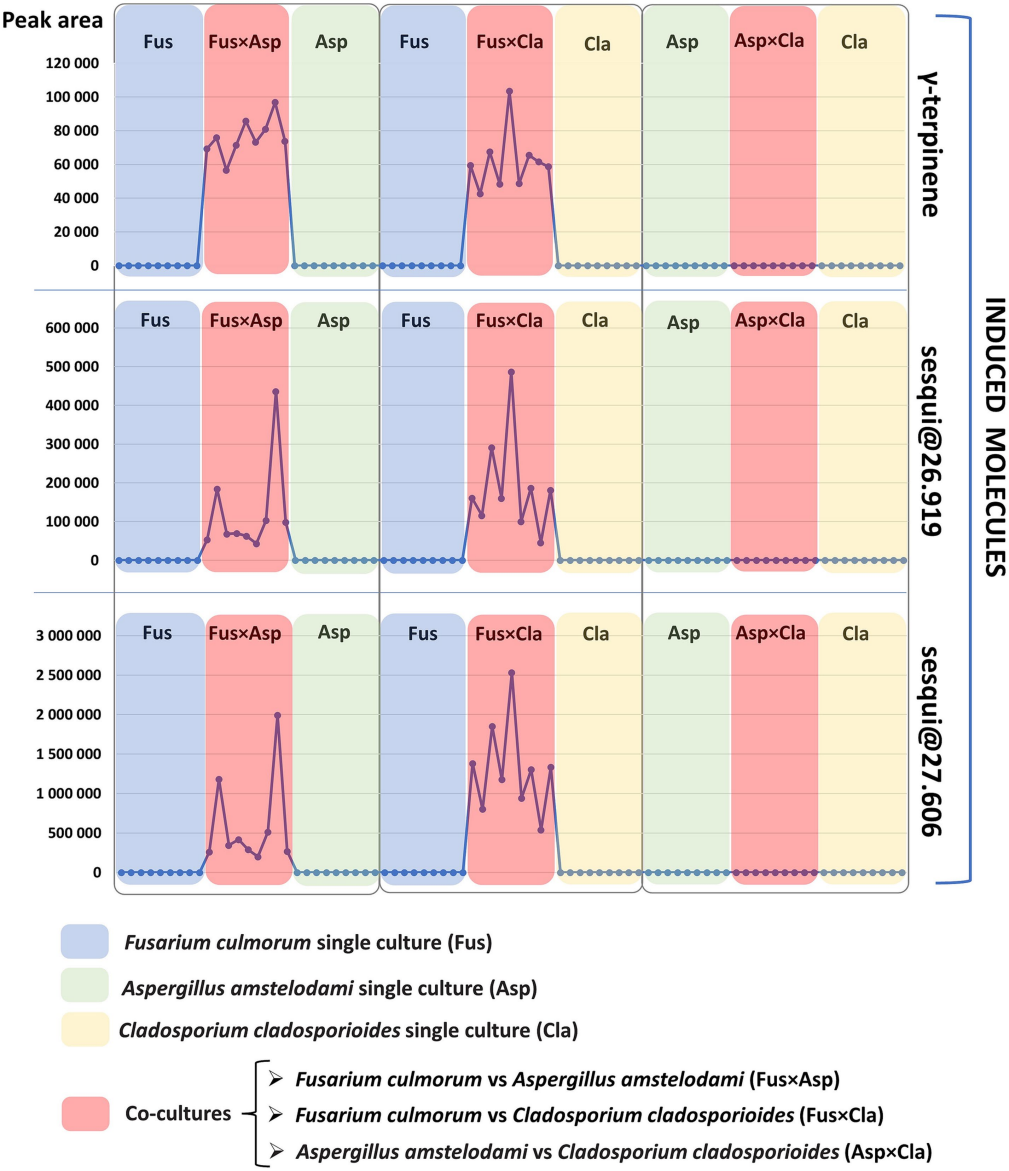
Induction pattern of the three volatile induced molecules (starting from the top of the figure: γ-terpinene, sesqui@RT26.919, and sesqui@RT27.606). Each single culture and co-culture sample is represented by a dot on the horizontal axis. The peak area detected for each volatile molecule is marked on the vertical axis. Co-cultures of *Fusarium culmorum* vs. *Aspergillus amstelodami*, *Fusarium culmorum* vs. *Cladosporium cladosporioides*, *Aspergillus amstelodami* vs. *Cladosporium cladosporioides* are indicated by the red color in this figure and are, respectively, referred to as FusxAsp, FusxCla and AspxCla. *Fusarium* (Fus), *Aspergillus* (Asp) and *Cladosporium* (Cla) single cultures are indicated by the following colors, respectively: blue for *Fusarium*, green for *Aspergillus* and yellow for *Cladosporium*. As evidenced here, induced molecules are detected only in co-cultures were *Fusarium culmorum* is one of the co-cultivated fungi: *Fusarium culmorum* vs. *Aspergillus amstelodami* (FusxAsp) and *Fusarium culmorum* vs. *Cladosporium cladosporioides* (FusxCla).

The presence of these specific features in the previously mentioned *Fusarium* co-cultures (FusxAsp and FusxCla), and their absence in the single cultures and in the AspxCla co-culture are depicted in Figure 3. In this representation, the variation in the absolute peak area (vertical axis) of these features across single cultures and co-cultures is displayed. Each point on the horizontal axis represents a single culture or co-culture (nine points for each single culture or co-culture represent replicates). For easier interpretation, co-cultures are colored in red, whereas single cultures of *Fusarium*, *Aspergillus* and *Cladosporium* are shown in blue, green, and yellow, respectively. As shown in Figure 3, the induced molecules consistently appeared in all the nine replicates of the *Fusarium*-related co-cultures (FusxAsp and FusxCla), while these molecules were not detected in any of the co-cultures of AspxCla and in any of the single cultures. This persistent induction is remarkable (Supplementary Figure S4), as the literature often reports cases in which molecules are predominantly expressed in co-cultures, but are still present, albeit at basal or lower levels, in single cultures (Bertrand et al., 2014b; Arora et al., 2020). The induction of these compounds suggests that certain silent biosynthetic gene clusters may be activated by the interaction of *Fusarium* with the other fungi.

Contrary to this persistent induction in all co-culture replicates of FusxAsp and FusxCla, very high variability was observed in terms of the peak area of the induced metabolites, especially for the two sesquiterpene compounds (Tables 1, 2 reports the average normalized peak area and standard deviation for the induced compounds). Given that biosynthetic gene clusters are activated in the context of fungal co-cultures to produce these induced molecules, it might be possible that epigenetic mechanisms contribute to the variability in metabolite expression as fungi dynamically prioritize resource allocation between growth and defense (Pfannenstiel and Keller, 2019; Pillay et al., 2022). Additionally, it can be hypothesized that fluctuations in metabolite production may play a role in preventing resistance in competitors. By varying their metabolite profiles, fungi may prevent other microorganisms from adapting to their chemical defenses. This variability in compound expression is well-described in the literature and explains the importance of conducting these studies with several biological replicates (Bertrand et al., 2014a), as was done in the context of this work.

**Table 1:** Normalized areas HS-SPME-GC-MS (*versus* internal standard, i.e., C13 in the dibutyl phthalate [DBP] solution) of the compounds emitted by single cultures and the co-cultures of *Fusarium culmorum* vs. *Cladosporium cladosporioides* (referred to as FusxCla in this table).

Exp	Tab	Compounds	Fus	Cla	FusxCla	
Ret index	Ret index		Average area	Average area	Average area	Std. dev.
1061	1060	γ-terpinene	//	//	0.02	0.005
1413	/	sesqui@RT26.919	//	//	0.05	0.03
1431	/	sesqui@RT27.606	//	//	0.35	0.17

The reported values are the average values of a different number of experiments (*n* = 9 for *Fusarium culmorum, Cladosporium cladosporioides* and the co-culture). Standard deviation values are also reported.

**Table 2:** Normalized areas HS-SPME-GC-MS (*versus* internal standard, i.e., C13 in the DBP solution) of the compounds emitted by single cultures and the co-cultures of *Fusarium culmorum* vs. *Aspergillus amstelodami* (referred to as FusxAsp in this table).

Exp	Tab	Compounds	Fus	Asp	FusxAsp	
Ret index	Ret index		Average area	Average area	Average area	Std dev.
1061	1060	γ-terpinene	//	//	0.02	0.003
1413	/	sesqui@RT26.919	//	//	0.03	0.03
1431	/	sesqui@RT27.606	//	//	0.15	0.14

The reported values represented are the average values of a different number of experiments (*n* = 9 for *Fusarium culmorum*, *Aspergillus amstelodami* and the co-culture). Standard deviation values are also reported.

The concurrent induction of *γ*-terpinene, sesqui@RT26.919, and sesqui@RT27.606 in two of the three co-cultures (FusxAsp and FusxCla) underscores a *Fusarium*-specific induction of these three molecules. This suggests that *Fusarium* could be responsible for eliciting these compounds in the two aforementioned co-cultures. Supplementary Figure S5 shows the detection of γ-terpinene, sesqui@RT26.919 and sesqui@RT27.606 only in the HS-SPME GC–MS metabolite profiles of FusxAsp and FusxCla.

Moreover, these results indicate the effectiveness of this experimental model in highlighting species-specific induction of volatile molecules. Thus, this methodology could be helpful in pinpointing the fungus responsible for the *de novo* production of metabolites in co-cultures.

Prior studies on the investigation of volatile interactions in microbial co-cultures have utilized solid-phase micro-extraction gas chromatography mass spectrometry (SPME-GCMS) (Spraker et al., 2014), or have employed gas chromatography ion mobility spectrometry (GC-IMS) (Speckbacher et al., 2021), or have been conducted in combination with ultrahigh-performance liquid chromatography coupled to high-resolution mass spectrometry (UHPLC-HRMS/MS) for the concomitant analysis of the non-volatile compounds involved in the interactions (Azzollini et al., 2018; Escudero-Leyva et al., 2023). However, despite these advances, the fundamental question of which fungus is responsible for the induction of specific volatile molecules has not been fully addressed in previous studies. The implementation of the proposed methodology could help to fill this gap and provide clearer insights into the identification of the species responsible for the volatile compound induction in co-cultures.

Additionally, the approach outlined in this study can be valuable for investigating fungal consortia and microbial interactions, particularly those of ecological and clinical relevance, such as those observed in the oral or lung mycobiomes (Belvoncikova et al., 2022; Fan et al., 2023). For example, it offers an effective approach for *in vitro* studies of fungi that comprise the lung mycobiome, where their presence and abundance may vary in correlation with the progression and severity of respiratory diseases such as asthma, chronic obstructive pulmonary disease (COPD) and allergic bronchopulmonary aspergillosis (ABPA) (Yii et al., 2017; Richardson et al., 2019; de Dios Caballero et al., 2022). These fungi are not merely passive colonizers; rather, they possibly engage in active biochemical competition, notably through the production and release of VOCs (Hung et al., 2015; El Ariebi et al., 2016). It is of great importance to gain an understanding of the VOC-mediated interactions and to identify the fungal species responsible for VOC production in competitive contexts, particularly during respiratory disease exacerbations when fungal communities often shift (Tiew et al., 2021; de Dios Caballero et al., 2022). Such insights could prove pivotal in the development of non-invasive diagnostic tools based on VOC monitoring via breath analysis (Sharma et al., 2023; Diefenderfer et al., 2024).

Furthermore, the findings from this study underscore the pivotal role *Fusarium culmorum* plays in biosynthetic processes when in co-culture with other fungal species. The chemical signals released during co-cultures may have specifically triggered the activation of silent biosynthetic gene clusters (BGCs), in *Fusarium culmorum*, leading to the production of the three induced volatile molecules mentioned above, including *γ*-terpinene. This induced compound may act as a signaling molecule, allowing interspecies interaction thorough non-contact mechanisms. Moreover, *γ*-terpinene may likely be produced by *Fusarium culmorum* as part of a defense mechanism against the other competing fungal species present in the co-cultures. Notably, the specific induction of γ-terpinene in its co-cultures with the other two fungal species highlights the need for further investigation into the biological activity of this compound. Understanding the role of γ-terpinene is crucial for gaining deeper insights into the ecological significance of *F. culmorum* in these interactions. To investigate this further, the effects of *γ*-terpinene on the growth of *Cladosporium cladosporioides* and *Aspergillus amstelodami* were evaluated.

### 3.3 Antifungal activity of *γ-*terpinene

For a better comprehension of the possible biological role of *γ-*terpinene in these interactions, the antifungal activity of this induced molecule was investigated against *Aspergillus amstelodami* and *Cladosporium cladosporioides*. Briefly, this bioassay consisted of a 9-cm Petri dish divided in two by a septum that allowed for the migration of only the volatile compound from one sector to another (Azzollini et al., 2018). In the first sector, the fungus was inoculated, whereas in the other sector, the volatile compound was injected into a filter paper. In this context, volatile compounds can migrate from one sector to another and inhibit fungal growth without direct contact with the fungal thallus.

**Figure 4:**
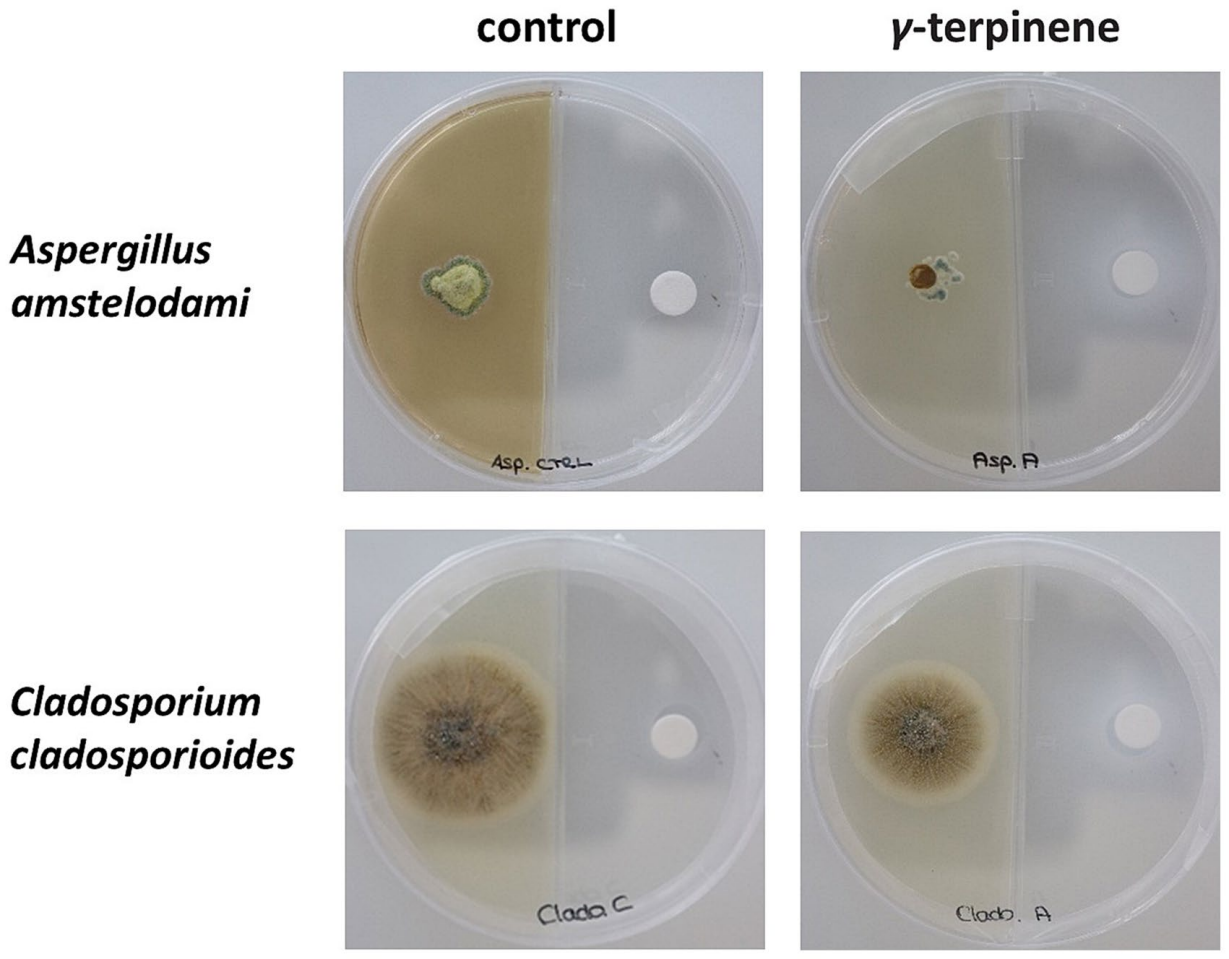
Antifungal bioassay performed using *γ*-terpinene at a concentration of 917 μL/L. The inhibition of the growth of the fungus *Aspergillus amstelodami* and *Cladosporium cladosporioides* could be noticed after 7 days (second column from left).

To assess the antifungal activity of *γ*-terpinene, a preliminary experiment was conducted in five replicates using a concentration of 917 μL/L (expressed as liquid volume of volatile compound per dish volume). Under these conditions, the growth of *Aspergillus amstelodami* and *Cladosporium cladosporioides* was inhibited after 7 days (Figure 4). To further evaluate the potency of *γ*-terpinene’s antifungal properties, the bioassay was executed in five replicates, also using two lower concentrations: 524 μL/L and 131 μL/L. Following a 7-day growth period, the mycelium growth inhibition occurred for both fungi also at the 524 μL/L concentration. Moreover, a slight inhibition of growth was observed for *Aspergillus amstelodami* at 131 μL/L (Supplementary Table S1). These findings, therefore, show the antifungal properties of *γ-*terpinene against both fungi *Aspergillus amstelodami* and *Cladosporium cladosporioides*. Our results, while consistent with previous studies highlighting the antifungal activity of γ-terpinene against *Candida albicans* (Rivera-Yañez et al., 2017) and endophytic fungi, such as *Botrytis cinerea* (Espinosa-García and Langenheim, 1991), contrast with the findings of a previous study where γ-terpinene did not exhibit any antifungal activity against *Cladosporium cladosporioides* (Agnieszka Wróblewska et al., 2019) when assessed using a diffusion disk method for fungal growth inhibition. This discrepancy in results may be attributed to differences in bioassay techniques and experimental conditions, with our setup being better suited to detect the inhibitory effect of γ-terpinene on fungal growth. In particular, our experimental design probably mimicked an environment in which γ-terpinene was more effective in exerting its antifungal activity. Factors such as the composition of the nutrient medium used for fungal growth and the exposure time to the tested molecule may have played a role in increasing the susceptibility of the fungus to γ- terpinene. Consequently, the observations in our study provide additional evidence of γ-terpinene’s broad-spectrum antifungal properties, extending its inhibitory activity to *Cladosporium cladosporioides* and *Aspergillus amstelodami.*

The production of volatile antifungal molecules during fungal co-cultures suggests a multifaceted ecological role of these induced molecules in microbial interactions. Their volatile nature allows them to function as chemical signals, facilitating interspecies communication without requiring direct physical contact (Morath et al., 2012; Schulz-Bohm et al., 2017). In such environments, volatile antifungal compounds like γ-terpinene may be synthesized as part of the biochemical arsenal to inhibit the growth of competing fungi. This inhibition may allow the producing organism to secure vital resources and establish dominance within its ecological niche, providing a competitive advantage (Hung et al., 2015; Quintana-Rodriguez et al., 2018). Additionally, induced antifungal molecules may influence growth patterns, and modulate ecological interactions within microbial communities of ecological as well of clinical significance, such as the human mycobiome (Belvoncikova et al., 2022; Hagihara et al., 2023). The role of antifungal VOCs, such as γ-terpinene, as a defense and signaling molecules underscores the importance of this type of compounds in fungal ecosystems. Moreover, it illustrates the evolutionary sophistication of fungi in developing chemical strategies to compete and communicate in dynamic microbial communities.

## 4 Conclusion

This study uniquely highlights the species-specific induction of two unidentified sesquiterpenes and of an antifungal volatile molecule, γ-terpinene, detected only in the *Fusarium*-related co-cultures FusxAsp and FusxCla. Additionally, it presents an effective methodology to investigate volatile fungal interactions and identify the species responsible for the induction of volatile molecules in co-cultures. Moreover, the proposed three-head-to-head co-culture strategy is broadly applicable and can be adapted to study co-cultures between bacteria and fungi (Moussa et al., 2020) allowing for a more comprehensive investigation of volatile interactions between different types of microorganisms (possibly part of the same microbiome or ecosystem). Detection of the specific induction of antimicrobial molecules within a microbial co-culture is of critical importance from ecological and microbiological perspectives. For example, from an ecological perspective, a better understanding of the interactions within a co-culture system can provide insights into how microbial communities function and coexist, thereby contributing to ecosystem stability and resilience (Braga et al., 2016; Gupta et al., 2021).

The methodology described in this study can be applied to investigate volatile interactions within microbial fungal consortia of ecological or clinical significance, such as those of the human mycobiome (Belvoncikova et al., 2022). It would be of interest to investigate whether VOCs produced during interactions of fungi associated with the oral or lung mycobiome can be detected not only through *in vitro* co-cultivation and GC–MS analysis, but also via electronic nose or via ambient mass spectrometry techniques in the breath of patients experiencing disease progression and exacerbations (Bregy et al., 2018; Finamore et al., 2019; VA et al., 2021). This could provide valuable insights into the progression of chronic lung diseases, where shifts in the microbial and mycobiome composition critically influence disease trajectory and outcomes (Tiew et al., 2021; Natalini et al., 2023; Scialò et al., 2023).

Furthermore, the presented methodology may be employed for the examination of the molecular dialog between disparate fungal taxa, thereby revealing various types of fungal interactions. For instance, it can highlight volatile molecules produced by fungi to recognize strains of the same species, or it can underscore which VOCs are produced between compatible or competing species. This exploration can also enhance our understanding of fungal mating processes through volatile compounds, shedding light on the intricate ways in which fungi communicate and interact at the molecular level (Bölker and Kahmann, 1993; Jones and Bennett, 2011; Wilson et al., 2023).

The proposed strategy may facilitate tackling these challenges, thereby enhancing our understanding of volatile molecular communication within *in vitro* fungal co-cultures and microbial consortia, an area that remains underexplored.

## Data Availability

Original GC-MS datasets are available in a publicly accessible repository: https://osf.io/f3gsv/?view_only=b60f211e21d24b2ca8113cc2298ad5af.

## Footnotes

^1^ https://mycoscope.bcis.ch/
