# Supplementary material for: A mass spectrometry-based strategy for investigating volatile molecular interactions in microbial consortia: unveiling a Fusarium-specific induction of an antifungal compound
Source: Front Microbiol. 2025 Feb 25;15:1417919. doi: 10.3389/fmicb.2024.1417919 (PMC11895703; doi:10.3389/fmicb.2024.1417919)
Supplement: Supplementary file 1 [file Data_Sheet_1.pdf]

Supplementary Material

A Mass Spectrometry-Based Strategy for Investigating Volatile Molecular Interactions in Microbial Consortia: Unveiling a *Fusarium*-specific Induction of an Antifungal Compound

Antonio Azzollini[1,2,3†*], Barbara Sgorbini[4†], Nicole Lecoultre[5], Carlo Bicchi[4], Jean-Luc Wolfender[1,2*], Patrizia Rubiolo[4*], Katia Gindro[5]

[1] School of Pharmaceutical Sciences, University of Geneva, 1211 Geneva, Switzerland.

[2] Institute of Pharmaceutical Sciences of Western Switzerland (ISPSO), University of Geneva, 1211 Geneva, Switzerland.

[3] Department of Laboratory Medicine and Pathology, Lausanne University Hospital (CHUV), 1011 Lausanne, Switzerland.

[4] Department of Drug Science and Technology, University of Turin,10125 Turin, Italy.

[5] Agroscope, Mycology group, 1260 Nyon, Switzerland.

*Corresponding Authors

[†]These authors contributed equally to this work

Table S1. Estimation of the antifungal potency of γ-terpinene. Table S1a shows the mycelium radius (mm) of *A. amstelodami* after seven-days at three different concentrations (917 µL/L; 524 µL/L and 131 µL/L) of the tested compound and for control samples. The letters _a, _b, _c, _d and _e indicate sample replicates (five replicates tested at each concentration). Mean and standard deviation values are also reported. In table S1b the mycelium radius (mm) of *C. cladosporioides* is reported (together with the mean and standard deviation values) at the above-mentioned concentrations and time point.

Table S1a

	mycelium radius (mm) - Control	mycelium radius (mm) - 917 µL/L	mycelium radius (mm) - 524 µL/L	mycelium radius (mm) - 131 µL/L
*A. amstelodami*_a	7.5	2	5	5
*A. amstelodami*_b	8	4.5	5.5	4
*A. amstelodami*_c	5	3.5	2.5	6.5
*A. amstelodami*_d	9.5	4	5	7.5
*A. amstelodami*_e	7.5	2.5	7.5	9
Mean	7.5	3.3	5.1	6.4
Std-dev	1.62	1.04	1.78	1.98

Table S1b

	mycelium radius (mm) - Control	mycelium radius (mm) - 917 µL/L	mycelium radius (mm) - 524 µL/L	mycelium radius (mm) - 131 µL/L
*C. cladosporioides*_a	20	17	17.5	22.5
*C. cladosporioides*_b	21.5	15	16.5	21
*C. cladosporioides*_c	22	15	19	17.5
*C. cladosporioides*_d	19	17.5	17.5	19
*C. cladosporioides*_e	21.5	15	17.5	22.5
Mean	20.8	15.9	17.6	20.5
Std-dev	1.25	1.24	0.89	2.21

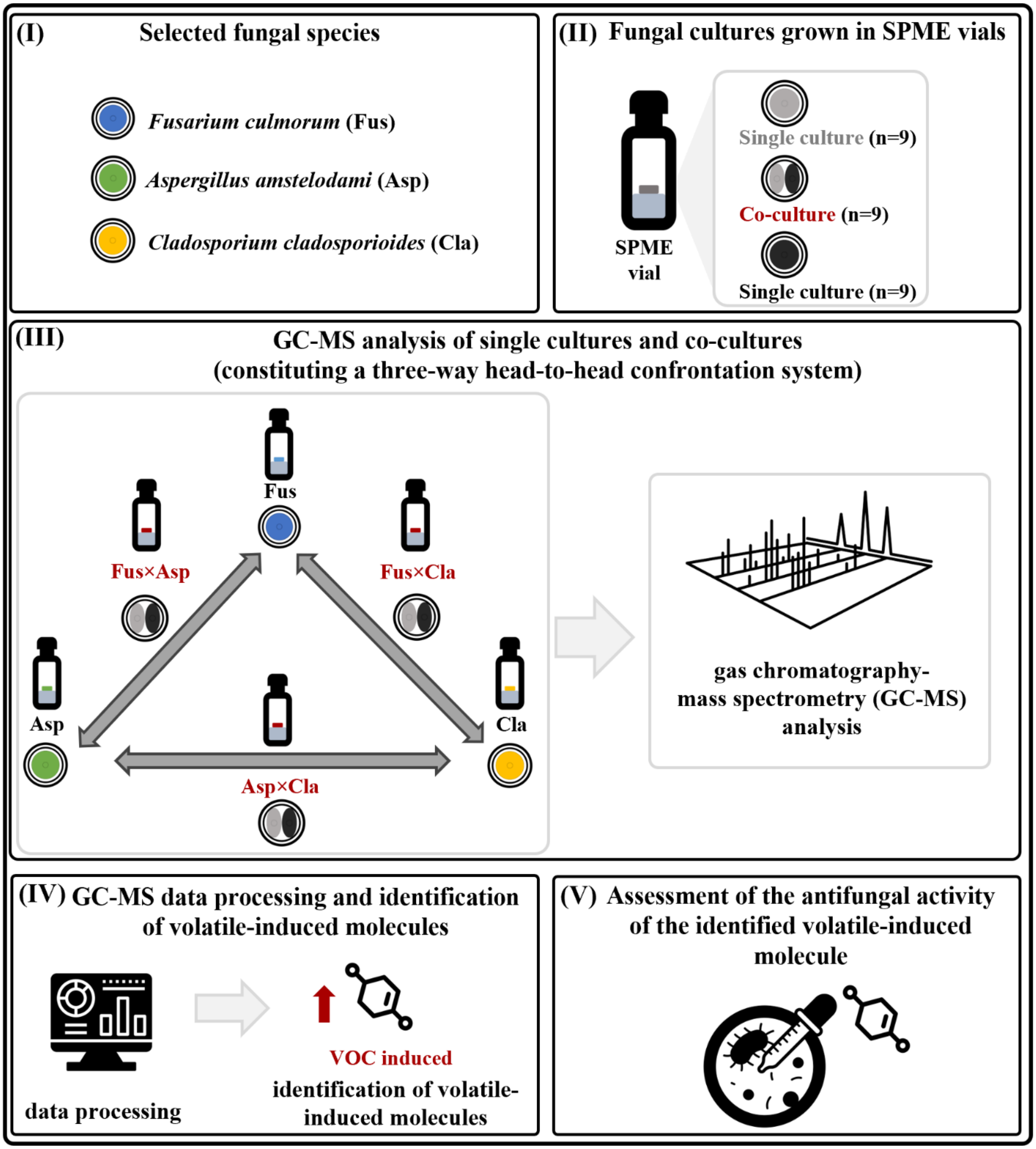

Figure S1 Overview of the experimental design of this study. (I) Three different fungal species were selected: *Fusarium culmorum*, *Aspergillus amstelodami* and *Cladosporium cladosporioides*. (II) Single cultures and co-cultures of these fungi were grown directly in SPME vials (nine replicates were prepared for both single and co-cultures). Pure cultures of *Fusarium culmorum*, *Aspergillus amstelodami*, and *Cladosporium cladosporioides* were prepared by placing 2-mm PDA plugs of fungal pre-cultures in the center of the headspace vial. The co-cultures were prepared by placing two 2-mm agar plugs of a pre-culture of the two different fungal species on opposite

sides of the headspace vial. (III) Three different fungal co-cultures (constituting a three-head-to-head confrontation system) were analyzed via gas chromatography-mass spectrometry (GC-MS): *Fusarium culmorum* vs. *Aspergillus amstelodami, Fusarium culmorum* vs. *Cladosporium cladosporioides*, *Aspergillus amstelodami* vs. *Cladosporium cladosporioides* (referred to as FusxAsp, FusxCla and AspxCla respectively) and of the corresponding single cultures (Fus, Asp and Cla). As each confrontation pairs one fungal species with another in the three head-to-head cocultures mentioned above, this confrontation setup is specifically designed to identify species-specific secondary metabolite induction. For example, metabolite induction in FusxAsp and FusxCla cocultures, with absence in AspxCla, may indicate a *Fusarium*-specific expression of the induced molecule. (IV) Following the GC-MS analysis, the data were processed to highlight features that were detected only in the co-cultures and not in the single cultures. Whenever possible the identification of a feature was confirmed by co-injection of a commercially available standard, thereby validating the identity of the induced volatile molecule in the co-culture system. (V) The induced and identified volatile molecule was then tested for antifungal activity.

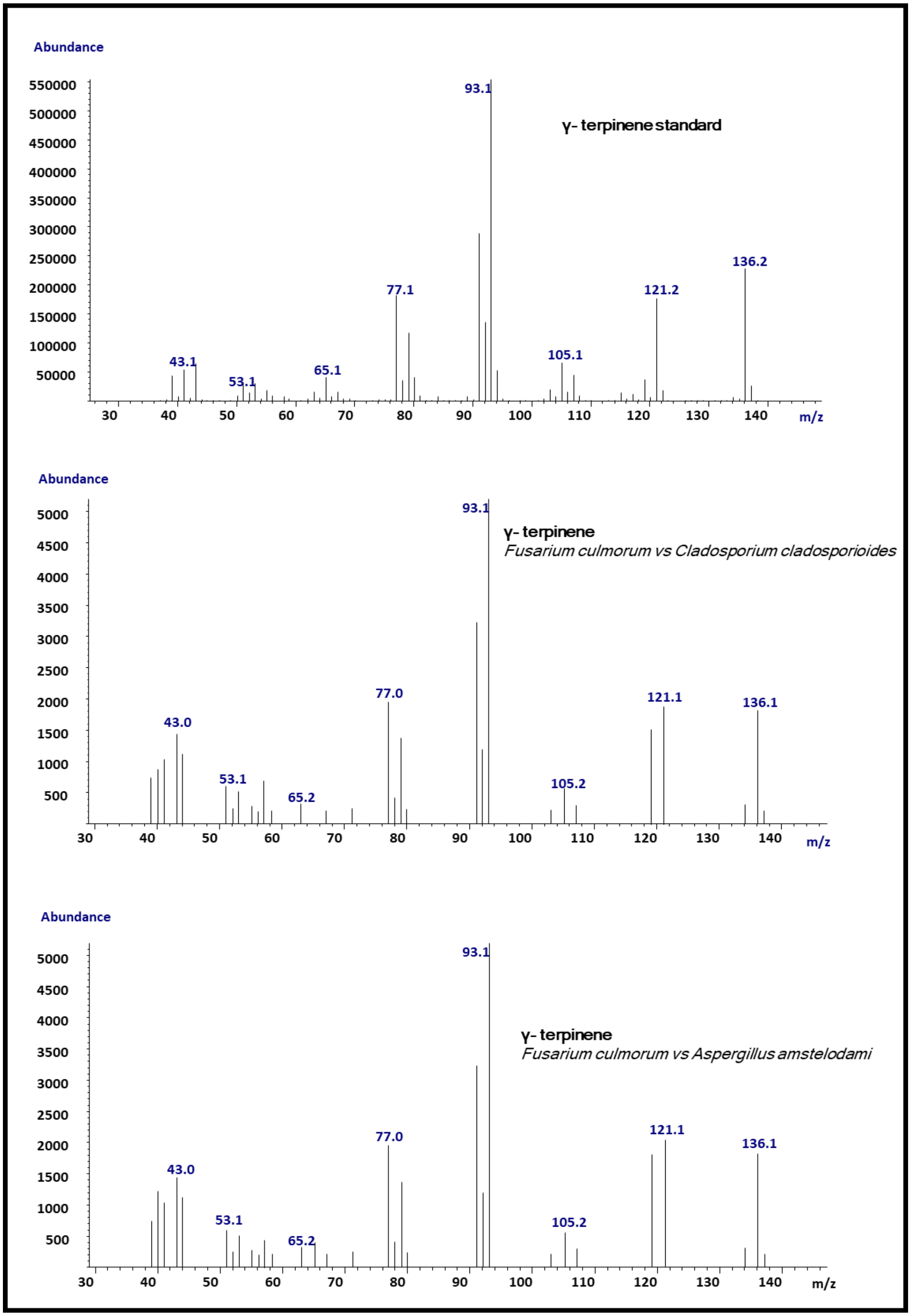

Figure S2 EI-MS fragmentation patterns of the *γ*-terpinene standard compound and the same compound detected in the *Fusarium culmorum* vs. *Cladosporium cladosporioides* as well as in *Fusarium culmorum* vs. *Aspergillus amstelodami* co-cultures.

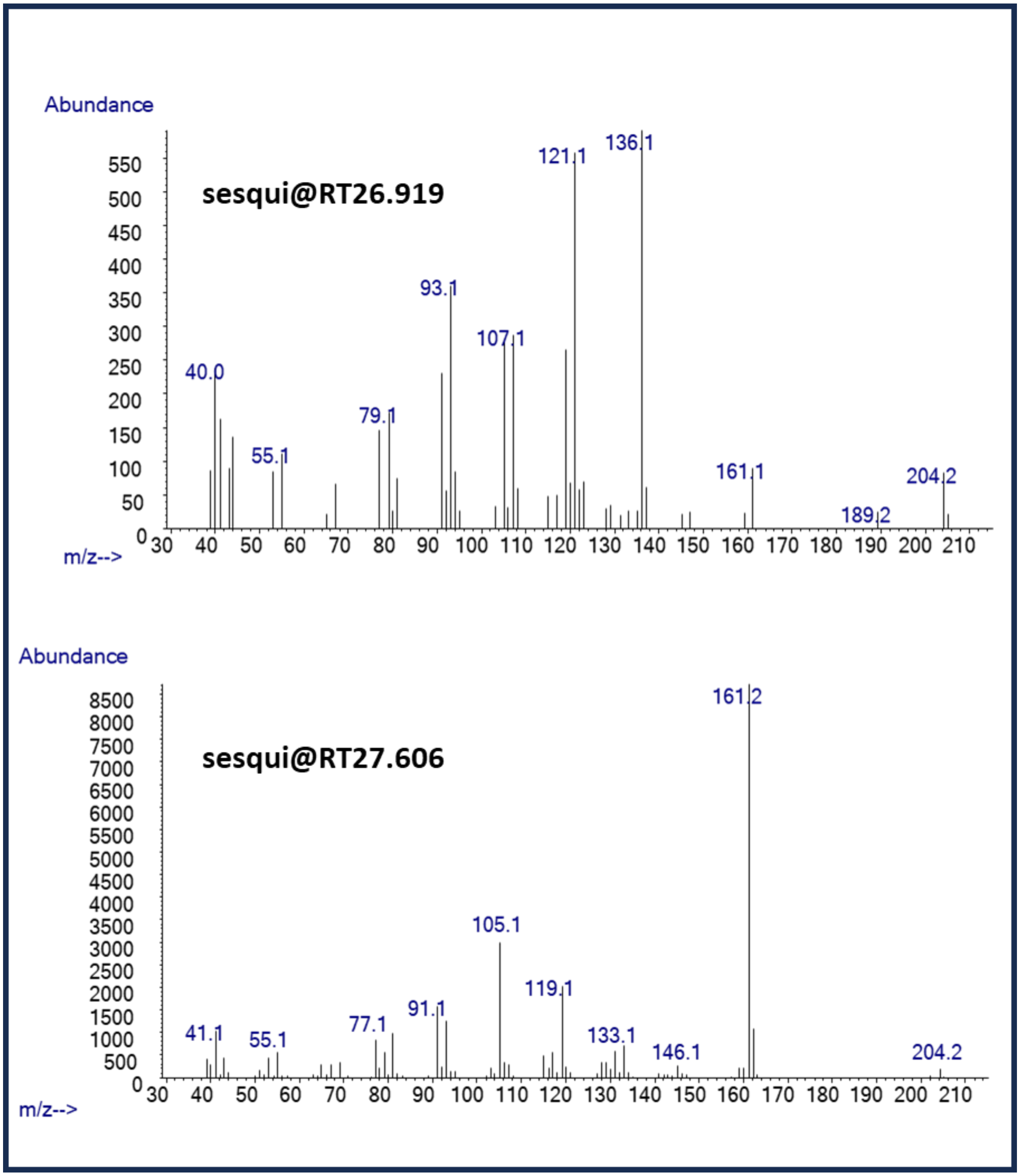

Figure S3 Electronic Impact-Mass Spectra of the two sesquiterpene compounds, referred to as sesqui@RT26.919 and sesqui@RT27.606 detected in the *Fusarium culmorum* vs. *Aspergillus amstelodami*, as well as the *Fusarium culmorum* vs. *Cladosporium cladosporioides* co-cultures.

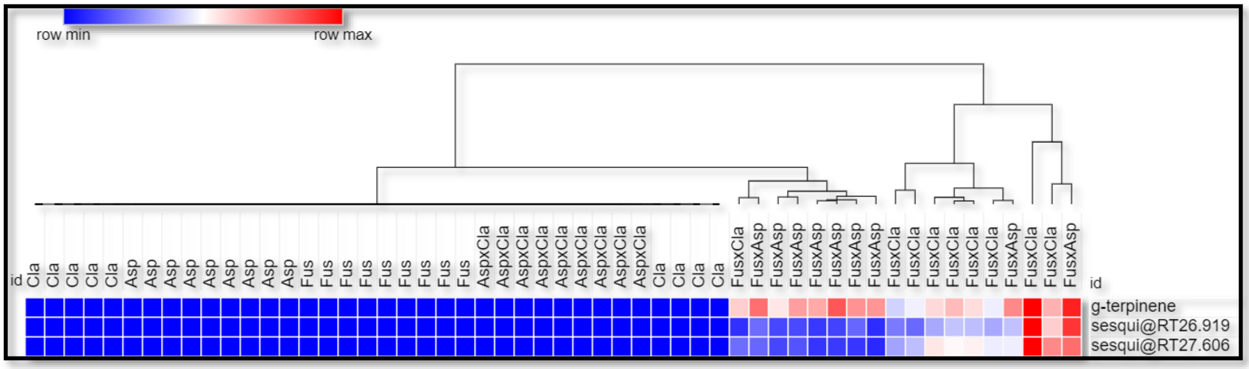

Figure S4 Heatmap with hierarchical clustering reporting volatile induced compound absolute peak area in the different single cultures and co-cultures. Every column represents a different sample. Each row represents one of the three different induced volatile compounds (γ-terpinene, sesqui@RT26.919 and sesqui@RT27.606) for which the maximum peak area is reported in red and the minimum in blue. On the x-axis, the codes read as follows: Cla represents single cultures of *Cladosporium cladosporioides,* Asp represents single cultures of *Aspergillus amstelodami,* Fus represents single cultures of *Fusarium culmorum,* AspxCla represents co-cultures of *Aspergillus amstelodami* vs. *Cladosporium cladosporioides,* FusxCla represents co-cultures of *Fusarium culmorum* vs. *Cladosporium cladosporioides* and FusxAsp represents co-cultures of *Fusarium culmorum* vs. *Aspergillus amstelodami*.

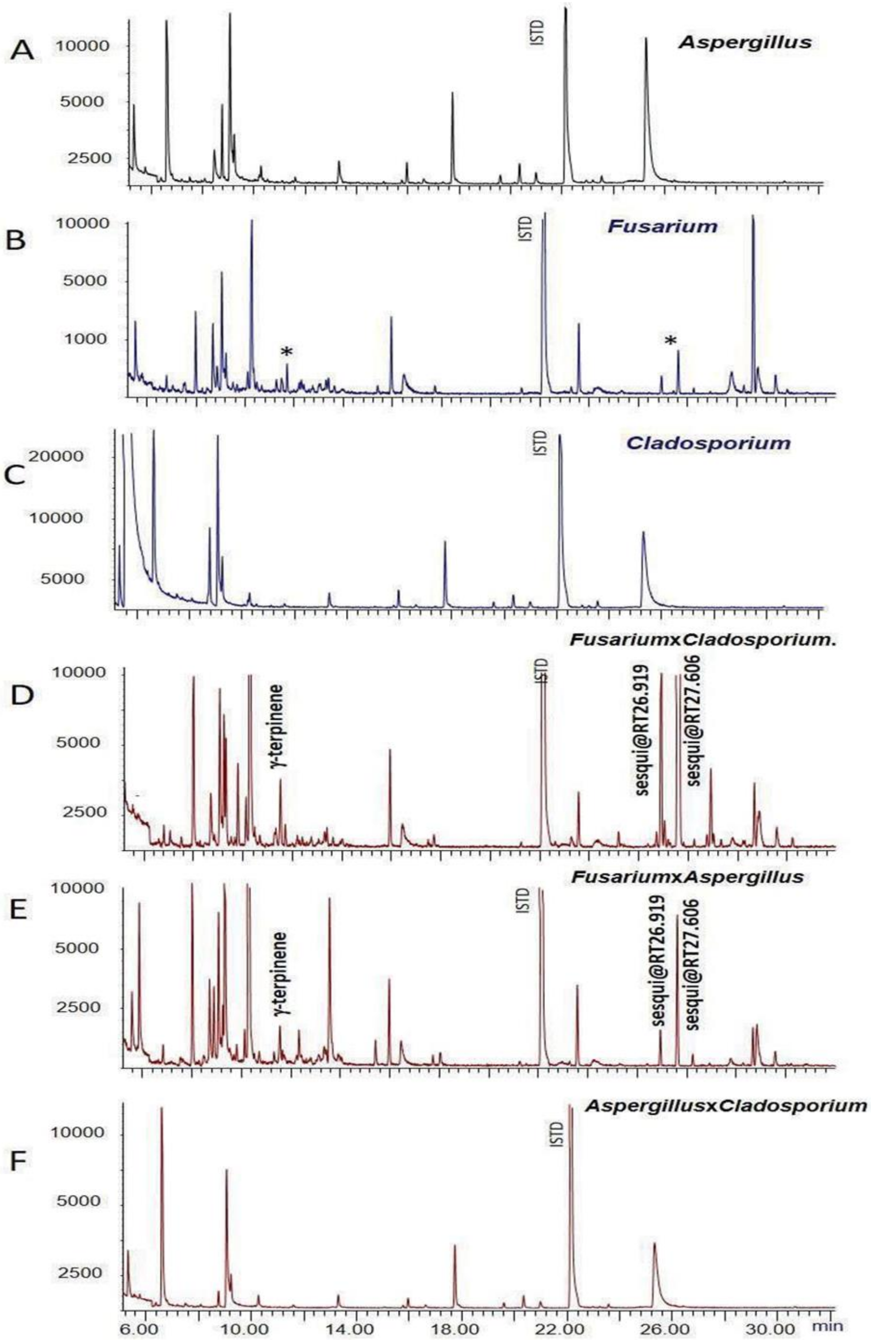

Figure S5 HS-SPME GC-MS metabolite profiles from the three single cultures and the three co-cultures analyzed in this work. The presence of γ-terpinene and the two unidentified sesquiterpene compounds (sesqui@RT26.919 and sesqui@RT27.606) can only be observed in the co-culture of (D) *Fusarium culmorum* vs. *Cladosporium cladosporioides* (*Fusarium*x*Cladosporium*) and (E) *Fusarium culmorum* vs. *Aspergillus amstelodami*, (*Fusarium*x*Aspergillus)*. These compounds were not detected neither in the co-culture of (F) *Aspergillus amstelodami* vs. *Cladosporium cladosporioides* (*Aspergillus*x*Cladosporium*), nor in the single culture of (A) *Aspergillus amstelodami*, (B) *Fusarium culmorum* and (C) *Cladosporium cladosporioides*. The symbol (*) in this figure indicates peaks that are unrelated to the induced molecules.
